# Overexpression of PP2A inhibitor SET oncoprotein is associated with tumor progression and poor prognosis in human non-small cell lung cancer

**DOI:** 10.18632/oncotarget.3818

**Published:** 2015-04-14

**Authors:** Hao Liu, Yixue Gu, Hongsheng Wang, Jiang Yin, Guopei Zheng, Zhijie Zhang, Minyin Lu, Chenkun Wang, Zhimin He

**Affiliations:** ^1^ Cancer Hospital and Cancer Research Institute, Guangzhou Medical University, Guangzhou, PR China; ^2^ Department of Microbial and Biochemical Pharmacy, School of Pharmaceutical Sciences, Sun Yat-Sen University, Guangzhou, PR China

**Keywords:** SET oncoprotein, non-small cell lung cancer, PP2A, FTY720

## Abstract

SET oncoprotein is an endogenous inhibitor of protein phosphatase 2A (PP2A), and SET-mediated PP2A inhibition is an important regulatory mechanism for promoting cancer initiation and progression of several types of human leukemia disease. However, its potential relevance in solid tumors as non-small cell lung cancer (NSCLC) remains mostly unknown. In this study, we showed that SET was evidently overexpressed in human NSCLC cell lines and NSCLC tissues. Clinicopathologic analysis showed that SET expression was significantly correlated with clinical stage (*p* < 0.001), and lymph node metastasis (*p* < 0.05). Kaplan-Meier analysis revealed that patients with high SET expression had poorer overall survival rates than those with low SET expression. Moreover, knockdown of SET in NSCLC cells resulted in attenuated proliferative and invasive abilities. The biological effect of SET on proliferation and invasion was mediated by the inhibition of the PP2A, which in turn, activation of AKT and ERK, increased the expression of cyclin D1 and MMP9, and decreased the expression of p27. Furthermore, we observed that restoration of PP2A using SET antagonist FTY720 impaired proliferative and invasive potential *in vitro*, as well as inhibited tumor growth *in vivo* of NSCLC cells. Taken together, SET oncoprotein plays an important role in NSCLC progression, which could serve as a potential prognosis marker and a novel therapeutic target for NSCLC patients.

## INTRODUCTION

Lung cancer is one of the leading causes of cancer deaths world-wide, and non-small cell lung cancer (NSCLC) accounts for approximately 80% of lung cancer diagnoses [1]. In spite of advances in developing more efficient surgical techniques and novel chemotherapeutic interventions, as well as targeted therapies, the long-term survival rate of NSCLC patients remains poor [2, 3]. Therefore, there is an urgent need for further understanding of the molecular mechanisms in lung cancer initiation and progression, and for identifying effective prognostic and diagnostic biomarkers and new therapeutic targets to improve the prognosis of patients.

Recent evidence suggests that there is a tight regulation of phosphatase and kinase activity in cancer cells [4-6]. Protein phosphatase 2A (PP2A), one of the main serine-threonine phosphatases, maintains cell homoeostasis by counteracting most of the kinase-driven intracellular signaling pathways [7]. PP2A has been shown to be genetically altered or functionally inactivated in many solid cancers and leukemia, and inhibition of PP2A activity or loss of some of its functional subunits is critical to promote cell transformation, tumor progression, and angiogenesis, which indicates that PP2A has tumor suppressive roles [7-9]. Consistent with its role as a tumor suppressor, PP2A plays a critical role in the regulation of cell-cycle progression, survival, and differentiation by negatively regulating the PI3K/AKT pathway [10, 11], and dephosphorylating and inactivating MEK1 and ERK family kinases [12, 13], or decreasing the stability and function of c-MYC transcription factors [14, 15]. Therefore, reactivation of PP2A activity base on its tumor suppressor properties is considered to be an attractive therapeutic strategy for human cancer treatment [16-18].

SET oncoprotein, also known as I2PP2A, is a potent physiologic inhibitor of PP2A [19]. SET protein directly binds with PP2Ac through its both N-terminus and C-terminus regions, and inhibits PP2A phosphatase activity [20]. The SET oncoprotein participates in the regulation of a wide variety of molecular processes [21-24]. Of importance, SET plays an oncogenic role modulating signaling pathways with high relevance in human cancer [25]. Several studies showed that SET was up-regulated in acute myeloid leukemia [26], head and neck squamous cell carcinoma (HNSCC) [27], colorectal cancer [25], and breast cancer [28]. Increased accumulation of the SET oncoprotein in these cancer cells accounts for decreased PP2A activity, and overexpression of SET promotes cell proliferation, survival, drug resistance, invasion and metastasis [25, 29-31]. Notably, restoration of PP2A activity through silencing of SET, or pharmacologic activation by SET antagonist FTY720 (fingolimod) resulted in reduced tumorigenesis *in vitro* and *in vivo* [32-36]. However, currently, little information has addressed the clinical significance of SET and the molecular mechanisms by which SET promotes the tumor progression of NSCLC.

In the present study, we found that SET expression was significantly increased in NSCLC cells and tissues, and was closely associated with tumor progression and poor prognosis. Knockdown of SET significantly inhibited the proliferation and invasion of NSCLC cells *in vitro* via activation of PP2A activities, and inhibition the AKT and ERK signaling pathway. Furthermore, SET antagonist FTY720 treatment increased PP2A activity, impaired cell proliferation, clonogenic potential and tumor growth of NSCLC cells *in vitro* and *in vivo*. Our data provide strong evidences that overexpression of SET was an independent prognostic factor for NSCLC patients, and inhibition of SET could be a promising therapeutic target in NSCLC.

## RESULTS

### SET is overexpressed and associated with poor prognosis in human NSCLC

**Figure 1:**
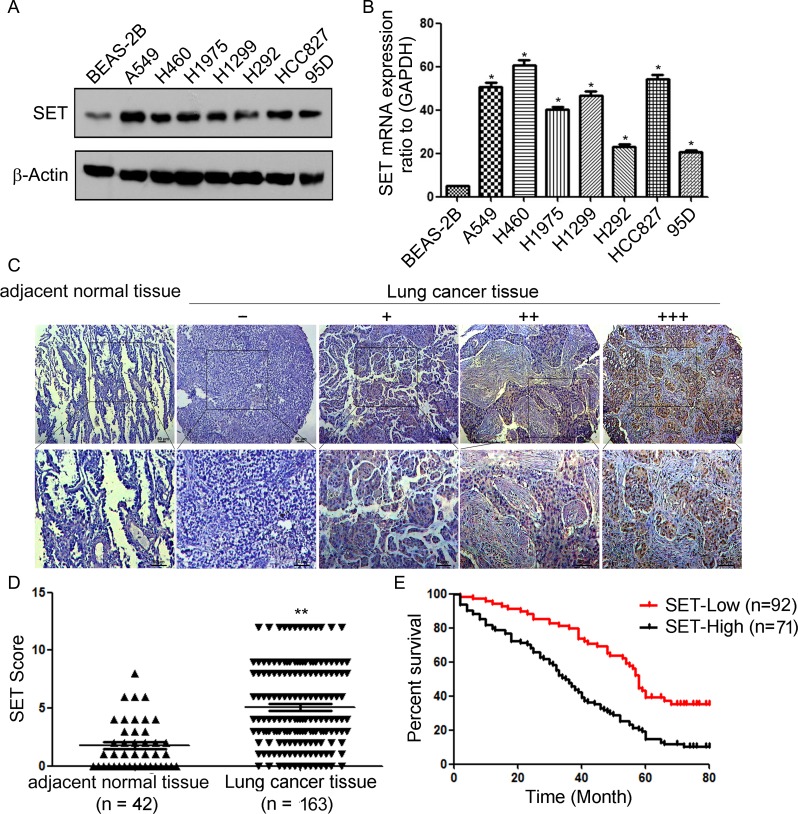
SET is overexpressed and associated with prognosis in NSCLC **A.** Western blot analysis of SET protein levels in normal human lung epithelial cell line BEAS-2B and NSCLC cell lines. **B.** RT-PCR analysis of SET mRNA levels in BEAS-2B and NSCLC cell lines. Experiments were repeated three times. *P < 0.05. **C.** Immunohistochemistry analysis of SET protein levels in NSCLC specimens and adjacent normal tissues. Representative immunohistochemical staining examples of SET protein expression in adjacent normal tissues and four different lung cancer tissues (Scale bar, 50 μm). The lung cancer tissue sections were quantitatively scored according to the percentage of positive cells and staining intensity as described in Materials and Methods. The percentage and intensity scores were multiplied to obtain a total score (range, 0–12), and the tumors were finally determined as negative (−), score 0; lower expression (+), score ≤4; moderate expression (++), score 5-8; and high expression (+++), score ≥9. **D.** SET expression scores in NSCLC specimens and adjacent normal tissues. **E.** Survival curves of NSCLC patients with low expression versus high expression of SET (*P* < 0.01, log-rank test). **P < 0.01 *vs*. adjacent normal tissues based on Student's *t*-test.

We first examined SET expression in a panel of 7 human NSCLC lines and 1 normal human lung epithelial cell line BEAS-2B. Western blot (Figure 1A) and RT-PCR (Figure 1B) showed high level of SET in nearly all of NSCLC lines compared with BEAS-2B. We then determined SET expression in clinical samples using immunohistochemistry analysis in 163 NSCLC specimens and 42 adjacent normal tissues and found that SET was overexpressed in 91.4% of tumor samples (149 of 163). While the adjacent normal tissues exhibited undetectable or low SET staining (Figure 1C and 1D). These results indicated that SET might be a critical molecule in lung cancer development.

Next, we analyzed the relationship between SET expression levels and clinicopathological characteristics. As shown in Table 1, no statistically significant correlations were observed between the expression of SET expression and gender, or age at diagnosis (P > 0.05). However, statistically significant correlations between high levels of SET expression were found with advanced TNM stage (*P* < 0.001), lymph node metastasis (*P* = 0.017).

**Table 1:** Correlation between the clinical pathologic features and expression of SET

Characteristics	Number of patients (n=163)	SET expression		*P*-value^a^
		Low (n=92)	High (n=71)	
Age				
<50	101	56	43	0.436
>50	62	36	28	
Gender				
Male	114	66	48	0.179
Female	49	26	23	
Clinical stage				
I	52	37	15	<0.001^b^
II	72	43	29	
III+IV	39	12	27	
Lymph node metastasis				
N0	106	74	32	0.017^c^
N1-3	57	18	39	

a X^2^ test.

b Comparing clinical stages I versus II, III-IV.

c Comparing Lymph node metastasis N0 versus N1-3.

To evaluate the prognostic value of SET expression in NSCLC, we divided the NSCLC patients into SET high and low expression groups based on a ROC curve cutoff score of 5. The expression of SET in each sample was subsequently classified as either high level (score ≥ 5) or low level (score < 5). Survival analysis revealed that NSCLC patients with high SET expression had poorer overall survival than those with low SET expression (*P* < 0.01; Figure 1F). Altogether, our present data suggested that SET is overexpressed in NSCLC and high level of SET expression is a prognostic predictor of progression and poor prognosis of NSCLC patients.

### Knockdown of SET suppress NSCLC cell proliferation and invasion

**Figure 2:**
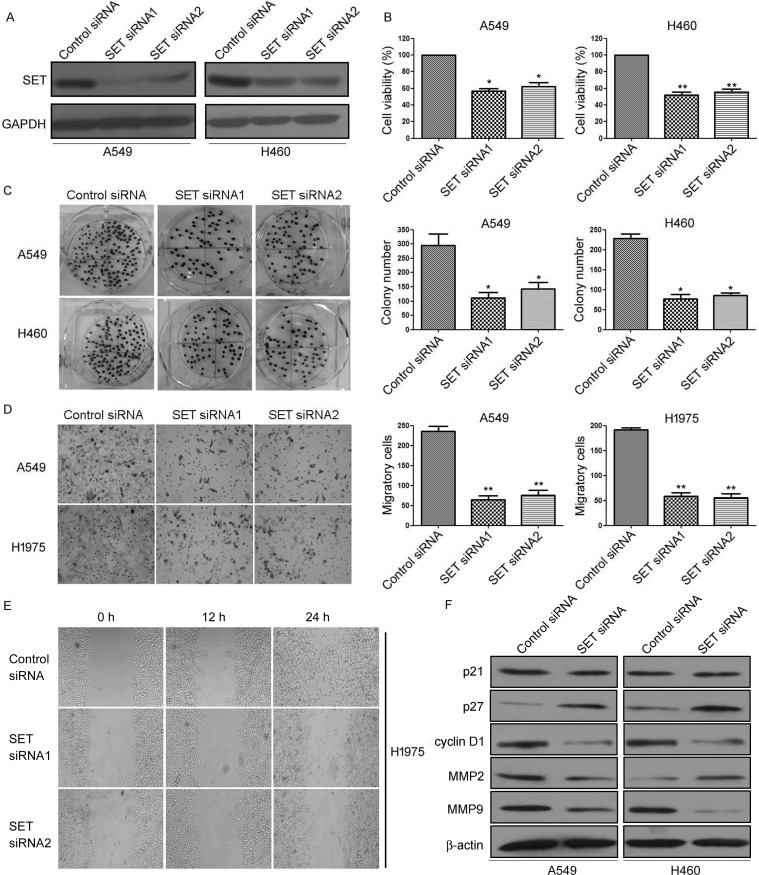
Inhibition of SET expression suppressed the proliferation and invasion of NSCLC cells *in vitro* **A.** A549 and H460 cells were transfected with SET-siRNA, SET protein expression was analyzed by using the Western blot method. GAPDH was employed as an inner control. **B.** A549 and H460 cells were transfected with SET-siRNA for 48 h, cell viability was evaluated by MTS assay. **C.** Colony forming ability of A549 and H460 cells transfected with SET-siRNA. (Left) Colonies were fixed with acetic acid-methanol (1:4) and stained with crystal violet. (Rgiht) The number of colonies was from three independent experiments. **D.** A549 and H1975 cells were transfected with SET-siRNA, cell invasive ability was detected by Matrigel invasion assay. (Left) Cells that adhered to the lower surface of the filtered were stained with hematoxylin. (Right) The number of migrated cells was from three independent experiments. **E.** The migratory speed of SET-siRNA-expressing A549 and H1975 cells was monitored through a scratch wound assay. **F.** A549 and H460 cells were transfected with SET-siRNA, the expression of p21, p27, Cyclin D, Cyclin E, MMP2, and MMP9 were analyzed by Western blot. Experiments were repeated three times. *P < 0.05, **P < 0.01.

To investigate the biological effect of SET deregulation on lung cancer cells, A549 and H460 cells were transfected with SET siRNA and performed using MTS, BrdU, and clonogenic assay. As shown in Figure 2, knockdown of SET (Figure 2A) significantly decreased cell viability (Figure 2B) and decreased the Brdu positive cells (Figure S1) in A549 and H460 cells compared to that of the control group. Furthermore, colony formation was also reduced after transfection with SET siRNA (Figure 2C).

In order to examine the role of SET in NSCLC cell invasion and migratory, we evaluated the effect of knockdown of SET on NSCLC cell invasion and migratory by transwell assays. As shown in Figure 2D, knockdown of SET drastically reduced the invasive ability of A549 and H1975 cells compared to that of control. Wound healing assay also showed that knockdown of SET significantly decreased the migratory speed of H1975 cells (Figure 2E).

Furthermore, we evaluated the effects of SET on the expression of several proliferative and invasive protein markers. Western blot analysis revealed that knockdown SET markedly enhanced the protein expression of cyclin-dependent kinase inhibitor p27 kip1, however suppressed the protein expression of Cyclin D1, as well as decreased the expression of matrix metalloproteinase 9 (MMP-9) (Figure 2F). Altogether, our results demonstrated that SET contributes greatly to the development of NSCLC cell proliferation and invasion.

### Inhibition of PP2A is essential for SET-induced proliferation and invasion

**Figure 3:**
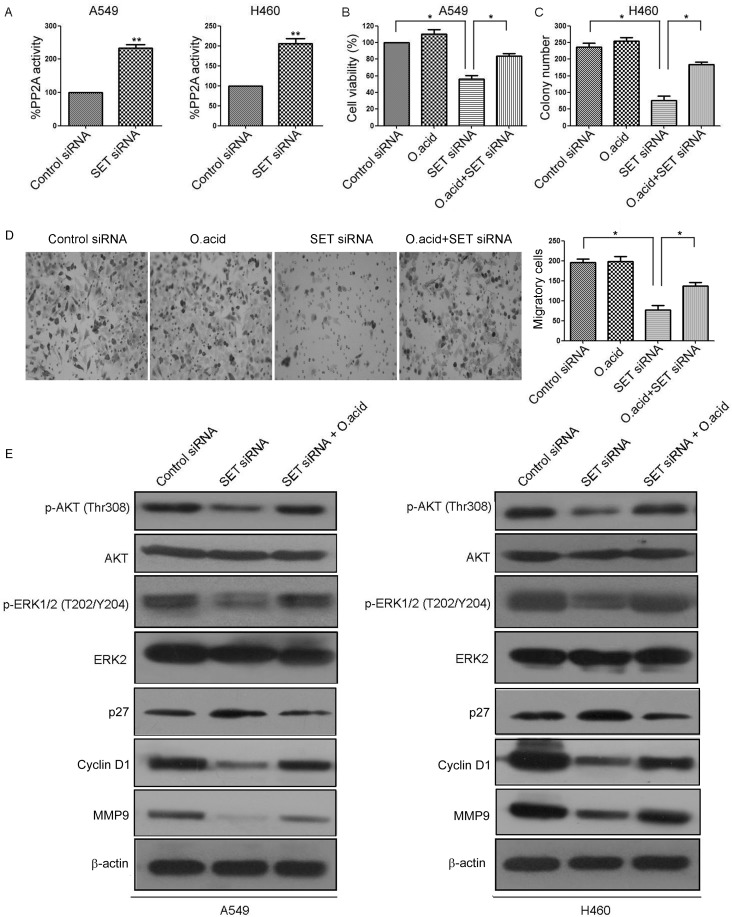
Inhibition of PP2A is essential for SET-induced proliferation and invasion **A.** A549 and H460 cells were transfected with SET-siRNA. PP2A activity was measured by PP2A immunoprecipitation phosphatase assay. **B.** A549 cells were transfected with SET-siRNA alone or in combination with 0.25 nmol/L okadaic acid for 48 h, cell viability was evaluated by MTS assay. **C.** Colony forming ability of H460 cells transfected with SET-siRNA alone or in combination with 0.25 nmol/L okadaic acid. **D.** A549 and H460 cells were transfected with SET-siRNA alone or in combination with 0.25 nmol/L okadaic acid, cell invasive ability was detected by Matrigel invasion assay. **E.** A549 and H460 cells were transfected with SET-siRNA alone or in combination with 0.25 nmol/L okadaic acid, levels of p-AKT, AKT, p-ERK, ERK, p27, Cyclin D, and MMP9 were analyzed by Western blot. Experiments were repeated three times. *P < 0.05, **P < 0.01.

The SET oncoprotein participates in the regulation of cellular molecular processes by inhibiting the tumor suppressor PP2A [19]. Then, we analyzed whether SET deregulation can alter the effects of the tumor suppressor PP2A in NSCLS cells. As expected, we observed PP2A activation after SET silencing (Figure 3A). To evaluate whether SET induced cell proliferation and invasion is due to inhibition of PP2A actiivity, we compared cell proliferation, colony formation and cell invasion in SET-siRNA expressing A549 cells and its control cells in the presence and absence of PP2A inhibitor okadaic acid (O.acid). Our results showed that O.acid significantly reversed the cell proliferation inhibited by SET siRNA (Figure 3B, Figure S2). Moreover, the number of colonies and invasive cells were also dramatically increased when SET-siRNA expressing cells were separately treated with O.acid (Figure 3C and 3D).

We next examined whether the inhibition of PP2A had any effect in the phosphorylation status of previously described PP2A targets. Consistent with previous reports about the effects of PP2A activation in other tumor models [12], knockdown of SET decreased phosphorylation of the PP2A targets AKT and ERK1/2 without affecting their expression levels (Figure 3E). Moreover, treatment of O.acid rescued AKT and ERK1/2 phosphorylation in SET-knockdown A549 cells, as well as in SET-knockdown H460 cells (Figure 3E). Of importance, treatment of O.acid rescued the expression of Cyclin D1 and MMP9 in SET-knockdown A549 cells, but increased the expression of p27. Similar results were also observed in H460 cells (Figure 3E). Taken together, these findings suggested that inhibition of PP2A is essential for SET-induced proliferation and invasion.

### Antagonism of SET using FTY720 inhibits NSCLC cell growth *in vitro* and *in vivo*

**Figure 4:**
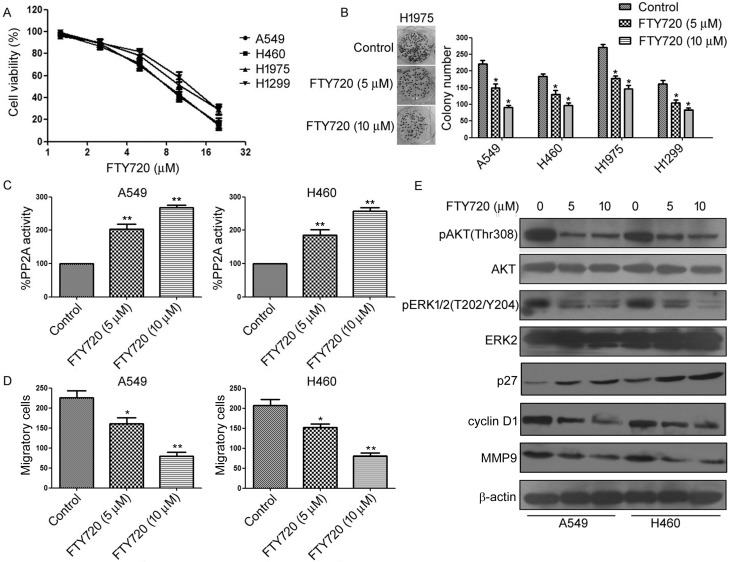
Antagonism of SET using FTY720 inhibits NSCLC cell growth *in vitro* **A.** A549, H460, H1299, and H1975 cells were treated with FTY720 at different concentration for 48 h, cell viability was evaluated by MTS assay. **B.** Colony forming ability of A549, H460, H1299, and H1975 cells treated with FTY720 at indicated concentration. **C.** A549 and H460 cells were treated with FTY720 at indicated concentration. PP2A activity was measured by PP2A immunoprecipitation phosphatase assay. **D.** A549 and H460 cells were treated with FTY720 at indicated concentration, levels of p-AKT, AKT, p-ERK, and ERK were analyzed by Western blot.

Recently, several studies reported that FTY720 and its precursors selectively bind to SET and increase PP2A activity [33, 34]. Therefore, we first evaluated the efficacy of FTY720 against NSCLC cells. We found that the treatment of FTY720 markedly increased PP2A activity in A549 and H460 cells (Figure 4A). Importantly, FTY720 treatment significantly inhibited NSCLC cell proliferation in a dose-dependent manner (Figure 4B). Reduction in viability of NSCLC cells after treatment with FTY720 also correlated with reduction of colony formation and cell invasion (Figure 4C and 4D). Moreover, treatment of FTY720 decreased the phosphorylation of AKT and ERK1/2, as well as the expression of Cyclin D1 and MMP9, but increased the expression of p27 (Figure 4E).

**Figure 5:**
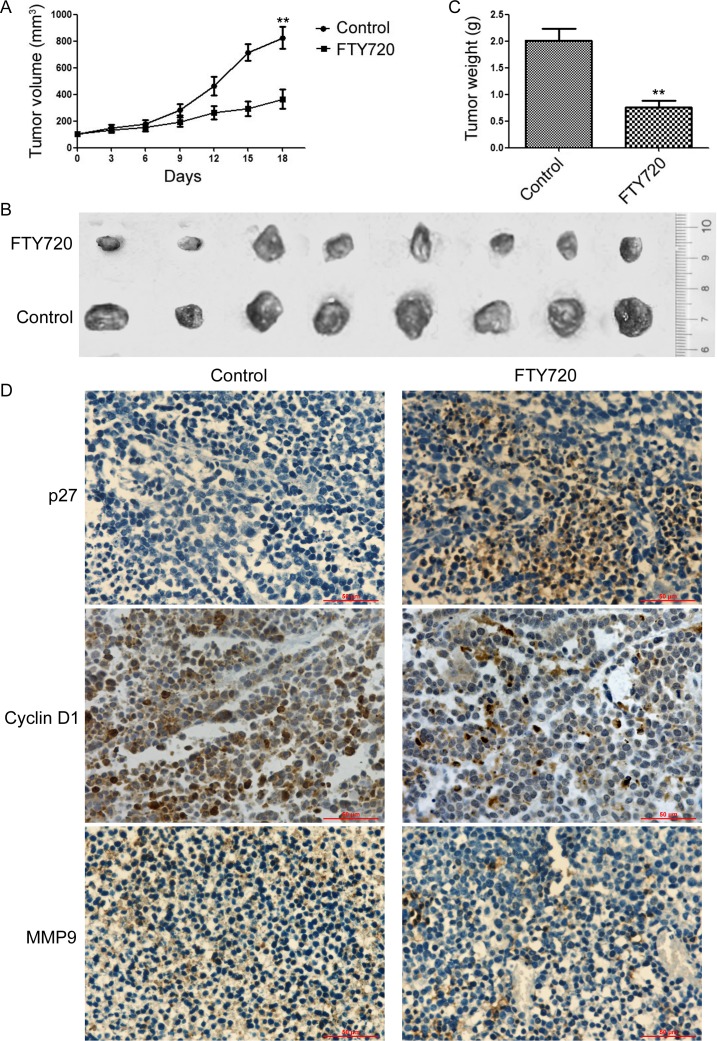
Antagonism of SET using FTY720 inhibits NSCLC cell growth *in vivo* A549 cells were injected to the right shoulder of nude mice and palpable tumors were allowed to develop for 7 days. Mice were randomly divided into two groups and treated intraperitoneally with DMSO, or FTY720 (3 mg/kg/day) for 18 days (*n* = 9). **A.** Tumor sizes were measured at every 3 days. At the end of treatment, tumor were excised **B.** and tumor weight were measured **C.**. Data are represented as means ±SD of each group.*P < 0.05, **P < 0.01. **D.** The tumor tissue sections were subjected to IHC detection of p27, Cyclin D1, and MMP-9 (Scale bars: 50 μm).

We further examined the ability of FTY720 to suppress tumor growth *in vivo*. The therapeutic potential of FTY720 against A549-xenografts generated in the flanks of SCID mice was determined after intraperitoneal administration of DMSO, or FTY720 (3 mg/kg/day for 18 days, n = 9). As shown in Figure 5A, FTY720 significantly inhibited the growth of tumor xenografts. Treatment with FTY720 resulted in a significant decrease in tumor volumes (263.8 ± 64.6) compared to control group (825.5 ± 81.2) (Figure 5B). The excised tumors from the control group weighed between 1.72 g and 2.25 g, whereas these from the FTY720-treated animals averaged ~ 0.68 g (Figure 5C). In addition, tumors derived from mice treated with FTY720 displayed higher PP2A activity (Figure S3) and the expression of p27 (Figure 5D), but lower levels of Cyclin D1, and MMP9 when compared with the control group (Figure 5D). Altogether, these results indicated that blocking SET/PP2A signaling by FTY720 could inhibit cell proliferation and invasive capability in NSCLC.

## DISCUSSION

In this report, our data provided the first evidences that SET is overexpressed in NSCLC and contributes to adverse clinical outcomes. We found that high expression of SET was correlated with advanced tumor stages and regional lymph node metastasis. We also showed that SET is deregulated in NSCLC cell and plays an oncogenic role promoting cell proliferation, colonosphere formation, and invasiveness, and impairing PP2A antitumor activities. Furthermore, our *in vitro* and *in vivo* results demonstrated that the pharmacologic activation of PP2A by SET antagonist FTY720 reduced cell viability and tumor growth of NSCLC cells

SET has been reported to be up-regulated in CML cells, resulting in decreased PP2A activity [32]. Similarly, SET overexpression in patients with several solid tumor, including colorectal cancer [25], head and neck squamous cell carcinoma (HNSCC) [27], colorectal cancer, and breast cancer [28], shows promising therapeutic implications and determines poor clinical outcome. In the present study, our data firstly demonstrated that SET expression was up-regulated in human NSCLC tissues and cultured NSCLC cells compared to that of normal lung tissues and normal lung epithelial cells. Of important, multivariate analysis indicated that SET may represent an independent prognostic biomarker for NSCLC patients.

Collected evidence supports the idea that SET is closely linked with tumor progression [35]. Notably, we found that high SET expression was strongly correlated with the clinical stages and lymph node metastasis in NSCLC, which suggested that SET is closely associated with NSCLC progression. It has been shown that SET knockdown was shown to negatively affect tumorigenesis in CML [32], and silence of SET with siRNAs in CRC cell lines induced a decrease in the cell growth and colonosphere formation ability in both number and size of the spheres formed [25]. Similarly, our results showed that SET knockdown significantly reduced cell proliferation properties and colony formation in NSCLC cells, indicated that SET deregulation is an alteration that plays a potential oncogenic role of SET in NSCLC. Furthermore, we also found that downregulation of SET inhibited NSCLC cell invasion. The effect of SET on promoting the invasiveness of NSCLC cells was verified the IHC results on the significant correlations between SET expression and lymph node metastasis in NSCLC patients.

PP2A is a key cellular serine-threonine phosphatase and is an essential tumor suppressor [7, 37]. It has been shown that deregulation of PP2A is a common event in lung cancer [38, 39]. Animal studies also showed that PR65α point mutations and deletions increase the incidence of lung cancer [40]. The SET oncoprotein is an important binding partner of PP2A with an inhibitory function [19]. Our finding that SET is frequently overexpressed in NSCLC cancers is novel and suggests that deregulation of SET as a possible contributing mechanism to inhibit PP2A, and overexpression of PP2A inhibitors may play an important role in the development of human NSCLC. Therefore, inhibition of SET could be a viable strategy to posttranslational target PP2A and inhibit tumor growth in NSCLC. Indeed, knockdown of SET significantly increased the PP2A activity, and decreased the level of PP2A targets pAKT and pERK, as well as decreased the expression of Cyclin D and MMP9. More important, inhibition of PP2A activity reversed these above effects. Hence, inhibition of PP2A is essential for SET-induced proliferation and invasion.

Previous work has demonstrated that the SET antagonist FTY720 activates PP2A, is cytotoxic to primary chronic lymphocytic leukemia cells, and decreases lymphoma xenograft tumor growth [41, 42]. Similar results from recently studies of treatment with another SET inhibitor OP449 showed activation of PP2A, as well as suppressed the tumor growth of cancer [43, 44]. To explore the therapeutic potential for PP2A activation through SET inhibition as an approach for NSCLC therapy, we treated NSCLC cells with FTY720 and measured tumor growth and oncogenic potential *in vitro* and in *vivo*. Consistent with the demonstrated ability of FTY720 suppresses tumor growth; we observed that FTY720 treatment resulted in decreased proliferation and colony formation *in vitr*o. Of importantly, FTY720 significantly suppressed tumor growth *in vivo.* Therefore, our results provided preclinical rationale for clinical development of FTY720 for the treatment of NSCLC.

In summary, the results of this study showed that SET overexpression is a recurrent molecular event that plays an oncogenic role in NSCLC, and inhibition of SET may provide an important therapeutic strategy for the treatment of NSCLC by targeting PP2A, and facilitate down-regulation of several PP2A-regulated targets including AKT and ERK kinase, p27, cyclin D1, and MMP9. Based on the functional characteristics and cancer-specific expression, our results indicated that SET oncoprotein could serve as a potential prognosis marker and a novel therapeutic target for NSCLC patients.

## MATERIALS AND METHODS

### Reagents and antibodies

FTY720 was purchased from Novartis (Basel, Switzerland). okadaic acid was purchased from Sigma-Aldrich (St Louis, MO, USA). MTS was from Promega (Madison, WI, USA). RPMI-1640 medium and fetal bovine serum were from GIBCO (Invitrogen, Carlsbad, CA, USA). Antibody against SET was purchased from proteintech (Chicago, IL, USA). Antibodies against Akt, phospho-Akt (Thr308), ERK2 and phospho-ERK (T202/Y204), Cyclin D1, p27, MMP2, MMP9, p21, and Cyclin E were purchased from Cell Signaling Technology (Beverly, MA, USA). Antibody against β-actin was obtained from Santa Cruz Biotechnology (Santa Cruz, CA, USA).

### Cell lines

BEAS-2B, A549, H1299, H460, H1975, and H292 cell lines were from American Type Culture Collection (Manassas, VA). HCC827 and 95D cell line were purchased from the Cell Bank of Chinese Academy of Sciences (Shanghai,. China). The cells were cultured in RPMI 1640 (Invitrogen, Carlsbad, CA) containing 10% fetal calf serum (Invitrogen), 100 IU/ml penicillin (Sigma, St. Louis, MO), and 100 μg/ml streptomycin (Sigma) in a humidified incubator of 5% CO_2_ at 37 °C..

### Patients and specimens

This study was approved by the Ethics Committee of Guangzhou Medical University. Primary tumor specimens were obtained from 163 patients diagnosed with non-small cell lung cancer who underwent complete resection in the Affiliated Tumor Hospital of Guangzhou Medical University between 2002 and 2009. Follow-up information was obtained from review of the patients' medical record.

### Immunohistochemistry

Surgically excised tumor specimens were fixed with 10% neutral formalin, embedded in paraffin, and 4-μm-thick sections were prepared. Immunostaining was performed using the avidin-biotin-peroxidase complex method (UltrasensitiveTM, MaiXin, Fuzhou, China). The sections were deparaffinized in xylene, rehydrated with graded alcohol, and then boiled in 0.01 M citrate buffer (pH 6.0) for 2 min with an autoclave. Hydrogen peroxide (0.3%) was applied to block endogenous peroxide activity, and the sections were incubated with normal goat serum to reduce nonspecific binding. Tissue sections were incubated with SET rabbit polyclonal antibody (1:250 dilutions). Staining for antibody was performed at room temperature for 2 h. Biotinylated goat antimouse serum IgG was used as a secondary antibody. After washing, the sections were incubated with streptavidin-biotin conjugated with horseradish peroxidase, and the peroxidase reaction was developed with 3,3′-diaminobenzidine tetrahydrochloride.

The intensity of SET staining was scored as 0 (no signal), 1 (weak), 2 (moderate), and 3 (marked). Percentage scores were assigned as 1, 1-25%; 2, 26-50%; 3, 51-75%; and 4, 76-100%. The scores of each tumor sample were multiplied to give a final score of 0-12, and the tumors were finally determined as negative (−), score 0; lower expression (+), score ≤4; moderate expression (++), score 5-8; and high expression (+++), score ≥9. Tumor sample scored (+) to (+++) were considered positive (overexpression). An optimal cutoff value was identified: a staining index of five or greater was used to define tumors of high expression, and four or lower for low expression. The cutoff value for SET was chosen based on a measure of heterogeneity using the log-rank test statistical analysis with respect to overall survival. All patients were followed up yearly, with the last follow-up being conducted in September 2014. Of the 163 patients, none of patients were lost to follow-up and seventeen patients were alive at the final follow-up in September 2014.

### Western blot analysis

Cells were lysed in cell lysis buffer containing 1% NP-40, 20 mM Tris-HCl (pH 7.6), 0.15 M NaCl, 3 mM EDTA, 3 mM EGTA, 1 mM phenylmethylsulfonyl fluoride (PMSF), 20 mg/mL aprotinin, and 5 mg/mL leupeptin. Equal amounts of protein were separated by SDS-PAGE and transferred to PVDF membrane (Millipore Corporation, Billerica, MA, USA). After blocking, the blots were probed with the indicated primary antibodies. After washing and incubating with secondary antibodies, the blots were visualized by ECL reagent (Millipore).

### RNA extraction and real-time RT-PCR

Total RNA isolated from cells was used E.Z.N.A.^®^ HP Total RNA Kit (Omega Bio-tek, Doraville, GA, USA). The reverse transcription was performed with the PrimeScript^®^ RT reagent Kit (TakaRa, Shiga, Japan). After mixing the resulting Complementary DNA template with SET, or GAPDH primers, respectively and TaKaRa SYBR^®^ Premix Ex Taq™, Quantitative Real-Time PCR reaction was performed on a ABI 7500 Fast Real-Time PCR System (Applied Biosystems, Foster City, CA). Gene-specific primer pairs used in this study are:

SET forward, 5′-AAATATAACAAACTCCGCCAACC-3′,

SET reverse, 5′-CAGTGCCTCTTCATCTTCCTC-3′;

β-actin forward, 5′- GGCCAGGTCATCACCATTG-3′,

β-actin reverse, 5′- GGATGTCCACGTCACACTTCA-3′.

β-actin was used as the reference gene. The relative levels of gene expression were represented as ΔCt-Ct gene-Ct reference, and the fold change of gene expression was calculated by the 2^−ΔΔCt^ Method. Experiments were repeated in triplicate.

### Cell viability assay

Cell proliferation was measured in triplicate wells by MTS assay in 96-well plates using the CellTiter 96 Aqueous One Solution Cell Proliferation Assay (Promega, Madison, WI, USA), following the manufacturer's indications.

### Colony formation assay

The cells were transfected with control or SET siRNA for 48 h. Thereafter, 300 cells were planted into 6-cm cell culture dishes. The medium was changed every four days. After two weeks of incubation under the conditions of 37°C and 5% CO2, colonies were fixed with acetic acid-methanol (1:4) and stained with crystal violet. Colonies containing more than 50 cells were counted.

### BrdU cell proliferation assay

Cells were treated with 0.03 mg/ml BrdU for 6–12 h, at 37 °C, fixed with 4% paraformaldehyde, washed in 0.1 M PBS (phosphate-buffered saline, pH 7.4) with 1% Triton X-100, and incubated with 1 M Hcl (hydrochloric acid) and 2 M Hcl. A borate buffer (0.1 M) was added and cells were blocked with 0.1 M PBS in the presence of 1% Triton X-100, 1.0 M glycine, and 5% normal goat serum, and incubated in the sequence with anti-BrdU and secondary anti-mouse HRP conjugated with diaminobenzidine (DAB).

### Wound healing scratch assay

Cells were grown as monolayers in triplicates in 12-well plates (2 × 10^5^) until confluent. Cells were created an artificial scratch wound, and cell debris was removed by washing with PBS. Cell migration was photographed and the width of the wound was measured.

### Cell invasion assay

Cell invasion was evaluated by a Boyden chamber assay. The polycarbonate filters (8 μm pore size, Corning Costar Corning Incorporated, NY, USA) were pre-coated with Matrigel Matrix (BD Biosciences), and reconstituted at 37 °C for 30 min to gel. Cells (1 × 10^5^) were suspended in 150 μl serum free RPMI 1640 and added into the upper chamber, while 600 μl of complete media was added to the lower chamber. After 24 h of incubation, the cells migrated through the matrigel and adhere onto the lower chamber was fixed in 4% paraformaldehyde for 20 min, stained with Mayer's hematoxylin (Sigma–Aldrich) and counted under upright microscope (five fields per chamber).

### PP2A activity assay

PP2A immunoprecipitation phosphatase assay kit (Upstate, Temecula, CA) was used to measure phosphate release as an index of phosphatase activity according to the manufacturer's instructions. Briefly, 100 μg protein isolated from cells was incubated with 4 μg anti-PP2A monoclonal antibody (CST) overnight. 40 μl of protein A agarose beads were added and the mixture was incubated at 4 °C for 2 h. Subsequently, the beads were collected and washed three times with 700 μl of ice-cold TBS and one time with 500 μl Ser/Thr Assay Buffer. The beads were further incubated with 750 mM phosphopeptide in assay buffer for 10 min at 30 °C with constant agitation. 100 μl of Malachite Green Phosphate Detection Solution was added and the absorbance at 650 nm was measured on a microplate reader.

### Small interfering RNA treatment

SET siRNA-1 (sc-43856), SET siRNA-2 (sc-156066) and Control siRNA (sc-37007) were purchased from Santa Cruz Biotechnology (Santa Cruz, CA, USA). Cells were transfected with 100 nM siRNA at 60% confluence in reduced-serum RPMI 1640. Lipofectamine 2000 (Invitrogen, Carlsbad, CA) was used for transfection following the manufacturer's protocols. Following transfection, the mRNA and protein levels were assessed 48 h later.

### Animal studies

All animal work was performed in accordance with protocols approved approved by the Animal Experimentation Ethics Committee of Guangzhou medical University. Immunodeficient mice, age matched and 4-6 weeks old, were used in assays for tumor growth in a subcutaneous xenograft model. 1×10^6^ A549 cells were transplanted into the right shoulder in mice. Mice were randomly divided into two groups (*n* = 9) and treated intraperitoneally with DMSO, FTY720 (3 mg/kg/day) for 18 days. Tumor growth was analyzed by measuring tumor length (L) and width (W) and calculating volume (V) through the formula, V = LW^2^/2.

### Statistical analysis

Statistical analysis was undertaken with SPSS 16.0 software. The χ2 test was used to examine possible correlations between SET expression and clinicopathologic factors. Unpaired two-tailed Student's t-test was used to determine the statistical relevance between groups. Survival curves were plotted using the Kaplan-Meier method and compared with the log-rank test. Receiver operating curve (ROC) was used to determine the optimal cutoff point based on progression end point for SET expression. *P* value of <0.05 was considered to indicate statistical significance.

## SUPPLEMENTARY MATERIAL FIGURES


