# Supplementary material for: Overexpression of PP2A inhibitor SET oncoprotein is associated with tumor progression and poor prognosis in human non-small cell lung cancer
Source: Oncotarget. 2015 Apr 14;6(17):14913–25. doi: 10.18632/oncotarget.3818 (PMC4558125; doi:10.18632/oncotarget.3818)
Supplement: Supplementary file 1 [file oncotarget-06-14913-s001.pdf]

# Overexpression of PP2A inhibitor SET oncoprotein is associated with tumor progression and poor prognosis in human non-small cell lung cancer

**Supplementary Material**

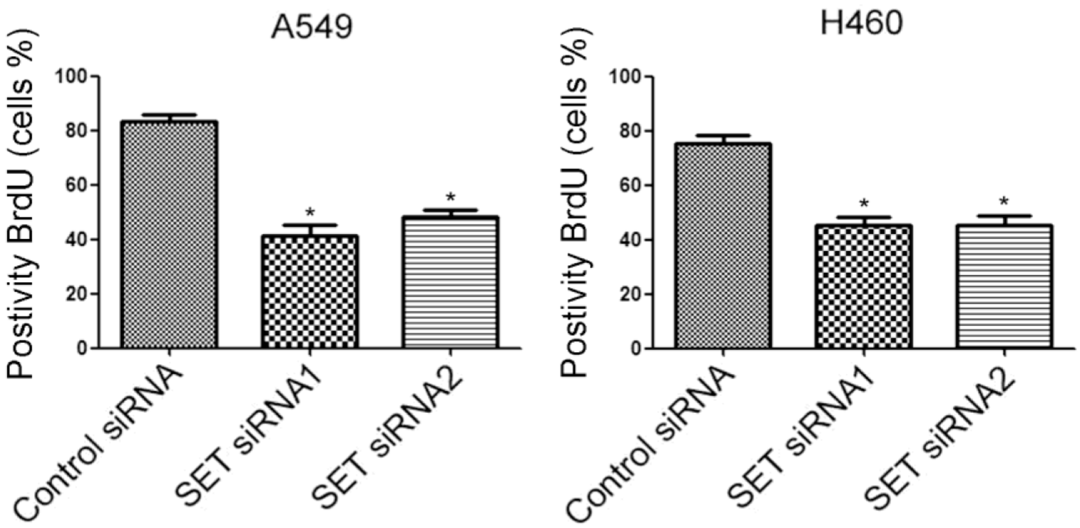


**Supplementary Figure S1.** Inhibition of SET expression suppressed the proliferation of NSCLC cells. A549 and H460 cells were transfected with SET-siRNA for 48 h, cell proliferation was evaluated by BrdU assay. Experiments were repeated three times. *P <0.05.

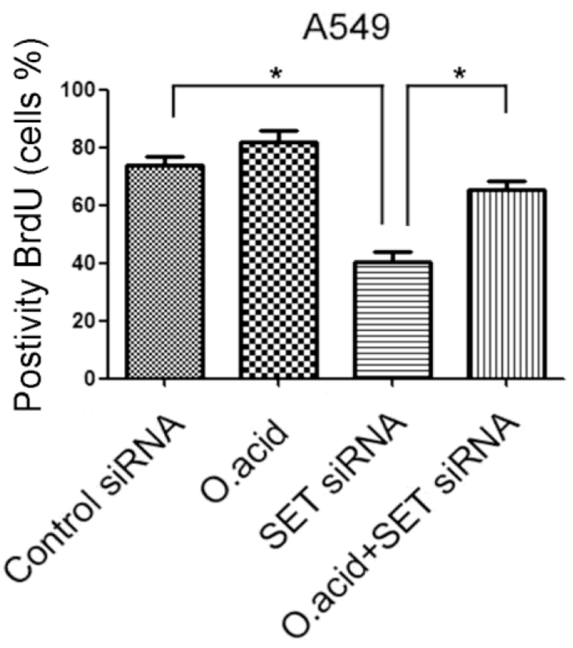


**Supplementary Figure S2.** Inhibition of PP2A is essential for SET-induced proliferation. A549 cells were transfected with SET-siRNA alone or in combination with 0.25 nmol/L okadaic acid for 48 h, cell proliferation was evaluated by BrdU assay. Experiments were repeated three times. *P <0.05.

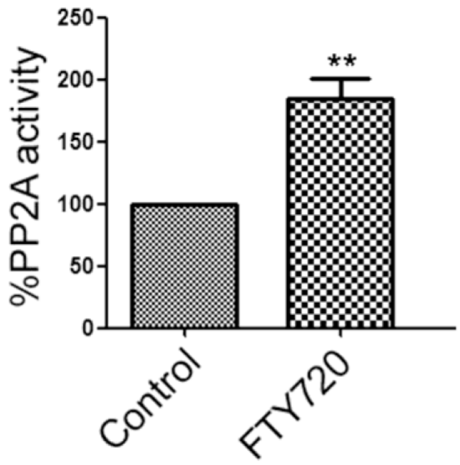


**Supplementary Figure S3.** Antagonism of SET using FTY720 increased PP2A activity *in vivo*. A549 cells were injected to the right shoulder of nude mice and palpable tumors were allowed to develop for 7 days. Mice were randomly divided into two groups and treated intraperitoneally with DMSO, or FTY720 (3 mg/ kg/day) for 18 days (n=9). At the end of treatment, tumors were excised, and PP2A activity was measured by PP2A immunoprecipitation phosphatase assay. Data are represented as means ±SD of each group. **P <0.01.
